# Worth working for: The influence of effort costs on teens’ choices during a novel decision making game

**DOI:** 10.1016/j.dcn.2019.100652

**Published:** 2019-04-30

**Authors:** Holly Sullivan-Toole, Samantha DePasque, Bailey Holt-Gosselin, Adriana Galván

**Affiliations:** a Department of Psychology, University of California, Los Angeles, 1285 Franz Hall, Box 951563, Los Angeles, CA, United States; b Graduate Program in Translational Biology, Medicine, and Health, Virginia Tech, Blacksburg, VA, 24061, United States; c Department of Psychiatry and Behavioral Sciences, Stanford University School of Medicine, 401 Quarry Road, Stanford, CA 94305, United States

**Keywords:** Effort, Reward, Adolescence, Adolescent, Development, Effort-based decision making, Effortful, Cost-benefit, Dual systems, Effort-discounting, Decision making, Reward response, Goal pursuit, Young adult, Physical effort, Motivation

## Abstract

Decision making requires consideration of both the benefits of a given choice and the costs, which can include risk, delay, and effort. Previous research has examined the developmental trajectory of adolescent decision making regarding risk and delay; however, the effects of effort on adolescent decision making remain largely unexplored. In the present study, we pilot tested a novel, developmentally-appropriate task designed to examine developmental differences in the willingness to expend effort during goal pursuit in adolescents (ages 13–16, n = 23) versus young adults (ages 18–23, n = 25). Self-reported reward responsivity correlated with task-related parameter estimates for effort and reward, providing evidence of task validity. Adolescents exhibited reduced sensitivity to physical effort costs compared to adults, effects which did not appear to be driven by differences in subjective task motivation or awareness of the effort requirements. These findings provide preliminary evidence that adolescence may be a time of increased willingness to expend effort during goal pursuit. Effort-based decision making is an understudied but exciting avenue for developmental research, as the willingness to engage in effortful pursuit of new experiences during adolescence may help to facilitate the path to independence.

## 1 Introduction

Behavioral and economic research has conceptualized decision making as a process of cost-benefit analyses, in which reinforcing outcomes are deemed worth pursuing only if their value outweighs the costs incurred to obtain them (Basten et al., 2010). Costs include investments of resources such as time, i.e., the delay between an action and an outcome; risk, i.e., uncertainty about whether an action might result in positive or negative consequences; and effort, i.e., physical or mental exertion (Floresco et al., 2008). When perceived as a cost, the exertion of effort must be motivated by a worthwhile “payoff,” i.e. a reward desirable enough to be worth working for. It has been argued that effort itself can be rewarding (Inzlicht et al., 2018), but, by and large, animal models of decision making have shown that increasing levels of reward are required to motivate increased levels of effort (e.g., Salamone et al., 2016a). Human research has demonstrated that when outcome values are held constant, lower-effort options are preferred (e.g., Sullivan-Toole et al., 2017) and that selection of higher-effort options is driven by increased reward magnitude and expected value (Treadway et al., 2009).

Adolescent decision making is a large and growing focus of study, with particular emphasis on peaking sensitivity to rewards and escalations in risk taking (see Chick, 2015; Galvan, 2010 for review of this literature). Adolescence is also a time of steadily decreasing levels of temporal discounting, meaning that while younger adolescents weigh delay costs heavily in their decisions, as they grow older, they become increasingly tolerant of delays in order to wait for higher-value rewards (e.g., Christakou et al., 2011; de Water et al., 2014; Steinberg et al., 2009). Unlike research on delay and risk, however, relatively little is known about the role of effort costs in adolescent decision making.

The present study introduces a novel effort-based decision making task, the alien blasting effort task. This task is premised on previous effort-based decision making tasks used in humans (Treadway et al., 2009) and in rodents (e.g., Hamill et al., 1999; Salamone et al., 1991) and offers several unique features that make it appropriate for youth and adult samples. Firstly, this task masks an effort-based decision paradigm in an “alien blasting video game” making it more engaging for younger participants while obscuring the research question. Second, a pre-task calibration procedure allows the task to be adjusted to a challenging, yet achievable, effort level for each individual. Thus, the task is adaptable for different developmental cohorts and it is possible to dissociate physical effort ability (which could vary with age) from motivation to exert effort. Third, the simplicity of the task makes it easy for even very young children to understand. The EEfRT (Treadway et al., 2009), another effort-based decision making task, which has been used extensively in adults, offers relatively complex decision trade-offs. Specifically, while decision options in the EEfRT partially confound effort with task difficulty and the probability of reward (contingencies adults can understand), the alien blasting task minimizes such effort confounds, as the individual task calibration renders unsuccessful trials uncommon across all effort levels. This not only allows the experimental variable effort to be studied in relative isolation, but also makes the task simpler for younger participants who are less proficient at integrating multiple decision factors. Fourth, this task utilizes points-based rewards (number of aliens hit) to incentivize performance. Previous research has shown that small differences in the number of points offered between conditions differentially motivates task performance and/or reward response in both adolescent and adult age groups (Colder et al., 2012; Hawes et al., 2017; Wang et al., 2017; Smith et al., 2016; Teslovich et al., 2014; van Leijenhorst et al., 2010a), suggesting that such abstract incentives are salient to both adolescents and to adults. Further, the use of such abstract incentives is ideal in research across developmental cohorts as it avoids the potential confound of monetary incentives having differential salience to different age groups (Davidow et al., 2016; Hawes et al., 2017).

Using this novel alien blasting effort task, the present pilot study tested the influence of effort and reward on preference and choice in adolescents and adults. Based on existing literature that indicates exaggerated sensitivity to reward during adolescence (Galvan, 2010; van Leijenhorst et al., 2010b), we hypothesized that increasing effort levels would impact adult decision making and preferences to a greater extent than it wouldin adolescents, who would be more inclined to base their choices on reward level regardless of the effort required.

## 2 Method

### 2.1 Participants

We recruited fifty-two healthy volunteers (n = 27 adolescents ages 13–16, n = 25 young adults ages 18–23). All volunteers who completed the study provided informed consent in accordance with the Declaration of Helsinki, with both youth and parental consent for participants under age 18. Four adolescent volunteers failed to obtain parental consent in accordance with IRB requirements and were not permitted to take part in the study, resulting in a sample size of 48 (23 adolescents, 13 female, mean age 15.17; 25 young adults, 14 female, mean age 20.28).

### 2.2 Effort task

**Fig. 1:**
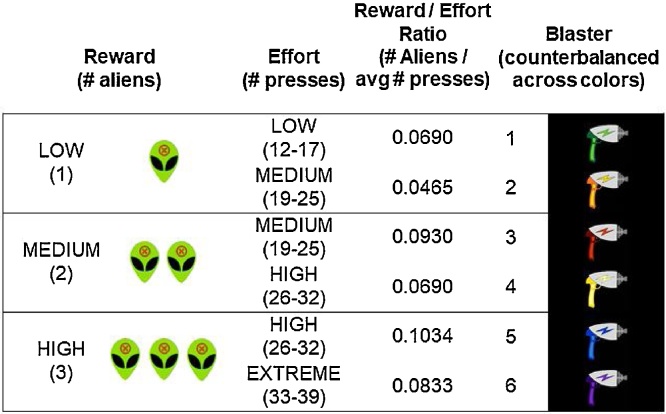
Blaster properties. Conditions were counterbalanced across colors. Increasing effort was required to attain greater reward, such that low effort was always paired with low reward, medium effort with low or medium reward, high effort with medium or high reward, and extreme effort only with high reward.

We developed a novel, adolescent-friendly paradigm that built upon a prior “alien blasting” physical effort task by Sullivan-Toole et al. (2017). The current task was adapted to investigate the influence of effort costs (i.e., number of button presses required) and reward magnitude (i.e., degree of task success afforded by a blaster option) on preferences and choices during the task. Based on established effort-based decision making paradigms in rodents (e.g., Hamill et al., 1999; Salamone et al., 1991) and in humans (Treadway et al., 2009), this task employed an objective manipulation of effort. Players were instructed to 'use six different blasters to stop groups of invading aliens' (Fig. 1). The task required participants to “charge” each blaster using repeated button presses to make it “fire,” and each blaster required a different average number of presses to achieve a successful charge. The task included four distinct effort levels, defined by the number of button presses required, and three levels of reward, defined by the number of aliens hit after a successful charge (reward and effort levels displayed in Fig. 1). Each blaster was deterministically associated with a specific effort requirement (low, medium, high, or extreme) and reward level (one, two, or three aliens), and successful charging reliably resulted in reward. Furthermore, each level of reward was paired with two blasters: one requiring relatively lower effort and one requiring relatively higher effort, which enabled us to probe effects of relative effort within each reward level, in addition to regression analyses examining the effects of the four effort levels. No two blasters were identical in terms of reward and effort contingencies; thus, a range of reward to effort ratios were represented across the six blasters (ratios displayed in Fig. 1).

### 2.3 Calibration procedure

To ensure that the task remained challenging yet achievable, the time limit for charging each blaster was calibrated to each participant’s speed on a training session prior to the task. After several practice trials, participants completed six calibration trials in which they were instructed to press as fast as they could. The average rate of pressing and the latency to begin pressing were each multiplied by 110% to set time limits that would be sufficiently challenging yet still yield high rates of success during the main task.

### 2.4 Task instructions

Participants were given no explicit instruction regarding the specific blaster contingencies; rather, they were instructed to pay attention to the different blasters, because ‘some might require more presses to charge and some might hit more aliens than others’. To establish hitting aliens as the rewarding element in the task, participants were instructed that the goal of the task was to blast as many aliens as possible. During a practice phase, participants experienced three sample trials with two separate practice blasters, which differed in both effort required and number of aliens hit. The practice blasters were distinct from those used in the main task.

### 2.5 Main task

**Fig. 2:**
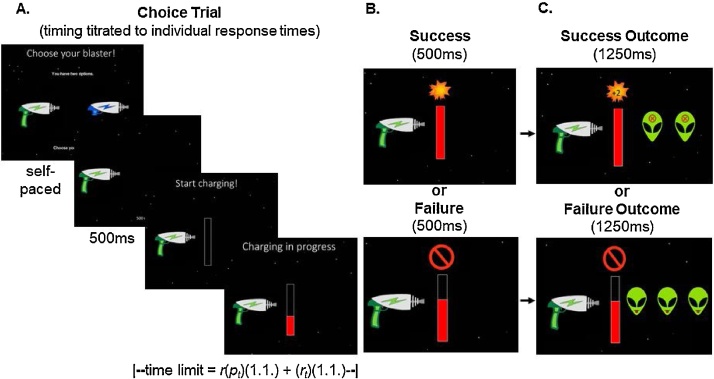
Task Schematic for Choice Trials. Participants completed five blocks of trials, in which they pressed “1” or “2” to pick up a blaster and pressed “x” rapidly to charge each blaster (A). During choice blocks (14 trials/block), trials presented an opportunity to choose between two blasters. The time limit for charging was individually calibrated based on performance during a pre-task calibration phase: time limit = *r*(*p_t_*)(1.1) + (*r_t_*)(1.1), where *r* = current number of press repetitions required, *p_t_* = average press rate during calibration, and *r_t_* = average response latency to begin charging during calibration. After participants successfully charged a blaster (B), they saw a feedback screen showing 1, 2, or 3 “blasted” aliens with an “x” on their foreheads, paired with the sound of one, two, or three blaster sounds, as well as “+1,” “+2,” or “+3″ to indicate the reward level (C, *top)*. When they did not succeed, they saw an image of three aliens teasing them with tongues stuck out (C, *bottom)*.

The task schematic is illustrated in Fig. 2. Participants first experienced the effort and reward contingencies of each blaster during a no-choice block (not shown in figure), in which only one blaster was presented per trial on either the left or right side of the screen (counterbalanced), while a grey “x” appeared on the opposite side. Participants were instructed to press a key (“1” for left, “2” for right) to “pick up” the blaster, and to charge the blaster by pressing “x” many times in rapid succession. During charging, each key press incrementally increased the “fill” level of the on-screen “charge bar” until it completely filled or until the individually calibrated time limit expired. No-choice blocks included 12 trials in random order (two repetitions of each blaster).

The initial no-choice block was followed by a 14-trial choice block, which included choice trials offering a choice between two blasters (˜10 choice trials per block), with no-choice trials intermixed (˜4 per block) to ensure continued exposure to less-preferred blasters. In total, participants completed five blocks of trials (two no-choice and three choice blocks). The block order followed a fixed sequence (no-choice, choice, no-choice, choice, choice), whereas trial order was randomized across the experiment. Participants completed a total of 36 no-choice trials (including those in no-choice blocks as well as those intermixed in choice blocks), ensuring that each blaster was used at least six times during the experiment. Additionally, there were 30 choice trials in which choices paired each of the blasters with each of the other blasters (each choice repeated twice). Choice trials were randomized across the three choice blocks.

### 2.6 Feedback screen

When participants successfully charged a blaster, they saw a feedback screen showing 1, 2, or 3 “blasted” aliens with an “x” on their foreheads (see Fig. 1 for illustration), paired with the sound of one, two, or three blaster sounds, as well as “+1,” “+2,” or “+3″ to indicate the reward level. When they did not succeed, they saw an image of three aliens teasing them with tongues stuck out.

### 2.7 Blaster preferences

Before and after performing the task, participants rated how much they would prefer using each of the six blasters in the game, on a scale from 1 (not at all) to 7 (very much). Before the task, they were shown the blasters and asked to base their decisions on anything they liked. Since the only feature differentiating the six blasters prior to game play was color, the ratings presumably reflected color preferences. After the session, they were asked to rate how much they would like to use the blaster based upon their experiences during the game. Adjusted preference ratings were used as the measure of preference. Adjusted preference ratings were calculated by subtracting the pre-task ratings from the post-task ratings in order to determine how game play influenced participants’ preferences for each blaster and to control for individual differences in preference that were unrelated to the game (e.g., an individual’s general preference for a particular color).

### 2.8 Post-task questionnaire

After the alien blasting game, participants completed a set of post-task questionnaires, which included the Dimensional Anhedonia Rating Scale (DARS; Rizvi et al., 2015), the Intrinsic Motivation Inventory (Ryan and Deci, 2000), and questions related to perceived effort and task reward salience. One participant did not complete the post-task questionnaire.

### 2.9 Dimensional anhedonia rating scale

The DARS (Rizvi et al., 2015) measures self-reported reward-related functioning across several domains and also yields an overall score. While the DARS was constructed as a measure of anhedonia (blunted reward response found within some psychiatric disorders), it is a good measure of general reward responsivity because experiences are rated in terms of reward on a scale from 1 (not at all) to 5 (very much), capturing normal to unhealthy levels of reward function. Additionally, this measure is appropriate for use across age groups because examples of rewarding experiences for each domain are self-generated (e.g., participants can list video games or bird watching as a personally rewarding experience), and thus it avoids the potential confound of age with rewarding experiences that have been pre-determined by a questionnaire. The overall score from the DARS was used to estimate general reward functioning in our participants.

### 2.10 Intrinsic motivation inventory

Participants were asked to report their level of motivation on the Intrinsic Motivation Inventory (Ryan and Deci, 2000), a multidimensional measure of subjective motivation during laboratory tasks. We administered three relevant subscales: interest/enjoyment (7 items, e.g., “I enjoyed doing this activity very much”), perceived competence (6 items, e.g., “I think I did pretty well at this activity, compared to other students”), and effort/importance (5 items, e.g., “I put a lot of effort into this”). Participants rated each item on a scale from 1 (not at all true) to 7 (very true), and average subscale scores were compared across the two age groups using independent samples t-tests to examine whether there were group differences in subjective motivation for the task.

### 2.11 Perceived effort

As a manipulation check, after the task, participants were asked how much effort was required to charge each blaster, on a scale of 1 (low/least effort) to 4 (extreme/highest effort). We were primarily interested in participants’ subjective response to the objective effort manipulation, rather than their accuracy for the exact press counts required. As such we used a rating scale with relative effort categories to measure the subjective experience of the effort manipulation. Within each age group, paired-samples t-tests were used to verify whether participants in both groups were able to accurately discern differences in categories of effort between blasters from adjacent effort levels (e.g., low vs. medium, medium vs. high, etc.). To compare accuracy of the category ratings between age groups, we also calculated accuracy scores by assigning each blaster an actual effort category value of 1 (low/least effort) through 4 (extreme/highest effort), subtracting actual effort category values from perceived effort category ratings, and averaging each participant’s accuracy scores across all six blasters.

### 2.12 Reward saliency

Two questions from the post-task questionnaire relate to the saliency of rewards used in the task (number of aliens hit). Participants were asked “During the task, how important was it for you to perform well (blast as many aliens as possible)?” and response options were provided on a 4-point scale: 1 (not important), 2 (slightly important), 3 (important) and 4 (extremely important). Participants were also asked “When your blaster hit the aliens, how satisfied did it make you feel?” and response options were provided on a scale from 1 (not satisfied) to 5 (extremely satisfied). Independent samples t-tests were used to compare reward saliency between the age groups.

### 2.13 Analyses

To establish whether adolescents and adults exhibited similar physical constraints on their task performance, we used an independent samples *t*-test to compare the response speed for the two age groups as measured during the calibration task.

To examine the relative weighting of effort costs versus reward outcomes in driving preferences for different blasters, we conducted two primary sets of analyses: one set of analyses examined effects of effort and reward on adjusted preference ratings, and a second parallel set of analyses examined the effects of effort and reward on choice behavior during the task. Adjusted preference ratings were calculated by subtracting pre-task preference ratings from post-task preference ratings. Choice behavior was defined as the number of times each blaster was selected during trials in which a choice between blasters was available.

### 2.14 ANOVA

For the ANOVAs, blasters were categorized based on reward level (low, medium, high) as well as relative effort level (low or high, based on whether the blaster was the relatively lower or higher effort level for a given reward level). Repeated measures ANOVAs with reward and relative effort as within-subjects factors and age group as a between-subjects factor examined effects of reward level, relative effort level, and age group on adjusted preferences/choice behavior. As the ANOVAs with dependent variables of adjusted preferences and choice behavior served as two parallel analyses testing the same overarching hypothesis (Rubin, 2017), Bonferroni correction for multiple comparisons was applied so that the adjusted critical alpha for these tests was *p* < 0.025. Effect sizes for F-statistics are reported using partial eta squared (ηp2).

### 2.15 Regression

As our sample size was less than 50, multi-level modeling was not used as our primary analysis strategy (Maas and Hox, 2005). Instead, multiple regressions were run at the subject-level in order to account for within-subject variation and the resulting regression parameters were used for subsequent analyses. However, multilevel models were also run (see for details see Supplementary Methods) and model results are presented in the Supplementary Results. Within-subject multiple regressions examined the extent to which preferences/choice behavior were driven by reward level or by effort level (4 levels of effort) for each participant. As these two regressions (adjusted preferences and choice behavior) served as parallel analyses testing the same overarching hypothesis (Rubin, 2017), Bonferroni correction for multiple comparisons was applied so that the adjusted critical alpha for these tests was *p* < .025. Additional subject-level regression models tested the degree to which preferences were driven by the ratio of reward to effort. For all regression analyses, unstandardized regression coefficients were used as individual difference variables to examine whether the weighting of effort versus reward level varied by age. For all t-tests performed on regression coefficients to compare between-group differences, effect sizes are reported using Cohen’s d (*d*).

### 2.16 Post-hoc correlation

As a post-hoc manipulation check, DARS scores were correlated with participants’ effort and reward regression coefficients to characterize the extent to which participants’ general reward functioning was associated with the effects of reward and effort in the alien blasting game. While these analyses were post-hoc, all of the tests conducted are reported in the manuscript and these tests were Bonferroni-corrected using an adjusted critical alpha of *p* < .025 to account for the two parallel sets of analyses run (on adjusted preferences and choice behavior) testing the same overarching hypothesis.

## 3 Results

### 3.1 Response rates

As expected, adolescents did not differ from adults in calibrated response rate, *t*(46) = .312, *p* = .757 (adolescent/adult *M* = 166.65/165.08, *SD* = 17.11/17.75), or total number of aliens hit, *t*(46) = 1.61, *p* = 0.114 (adolescent/adult *M* = 67.57/68.20, *SD* = 1.62/1.08, indicating no age-related differences in physical performance or ability to succeed at the task. We did not expect any between-group differences in these measures as the purpose of the pre-task calibration procedure was to ensure each individual’s task was set to be challenging, yet achievable, based on their own performance when instructed to “press as fast as you can”.

### 3.2 Dars

Adolescents had significantly lower scores (higher anhedonia) on the overall DARS compared to adults *t*(45)= -2.47, *p* = .017 (adolescent/adult *M* = 69.23/75.60, *SD* = 10.01/7.64). There were no outliers driving this between-group difference and the minimum scores within each age group (adolescent min = 50; adult min = 54) were well-above the range of DARS scores previously reported among a sample of depressed adults (range of DARS score in depressed adults = 1–44; Rizvi et al., 2015). It should be noted here that clinical norms for the DARS in an adolescent sample have not been published.

### 3.3 Task motivation

On the three subscales of the Intrinsic Motivation Inventory, rated on a scale from 1 (not at all) to 7 (very), both age groups expressed similar subjective motivation to perform well during the task, as evidenced by similar levels of: interest/enjoyment, *t*(45)=-.28, *p* = .781 (adolescent/adult *M* = 4.32/4.43, *SD* = 1.35/1.35); perceived competence, *t*(45)=-.81, *p* = .425 (adolescent/adult *M* = 5.12/5.35, *SD* = .73/1.17); and effort/importance, *t*(45)=-.55, *p* = .591 (adolescent/adult *M* = 5.05/5.25, *SD* = 1.40/.98).

### 3.4 Reward saliency

Both adolescent and adult participants rated the importance of blasting aliens as very close to “important” (score of 3 on a 4-point scale) and there was no difference between the age groups in their ratings of importance (*t*(45) = 4.21, *p* = .68 (adolescent/adult *M* = 2.86/2.96, *SD* = 0.83/0.74). Similarly, both adolescent and adult participants rated the satisfaction of blasting aliens close to “neutral” (the middle option on a 5-point scale) and there was no difference between the age groups in their ratings of satisfaction (*t*(45) = 2.85, *p* = .78 (adolescent/adult *M* = 3.18/3.28, *SD* = 1.05/1.28).

### 3.5 Perceived effort

**Fig. 3:**
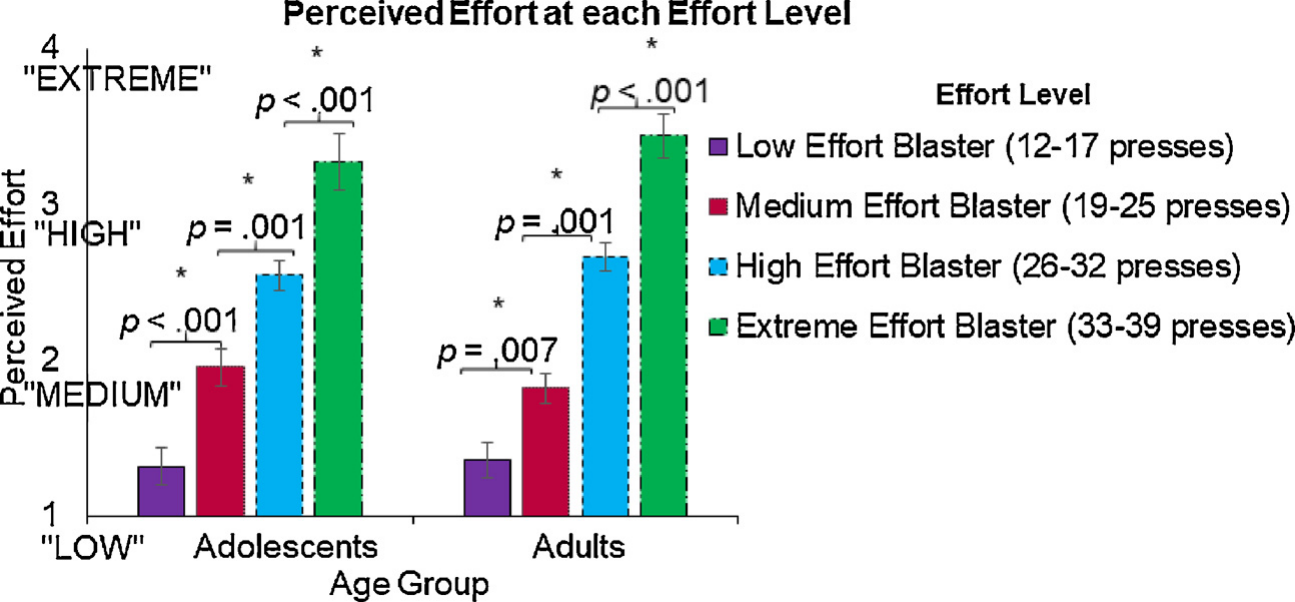
Perceived Effort Category Rating for each Effort Level. Post-task ratings of perceived blaster effort category indicated that participants in both age groups were able to accurately differentiate between blasters from different effort categories.

Both age groups were able to differentiate blasters across the four different effort level categories (Fig. 3). Accuracy for the perceived effort categories did not differ between age groups (*t*(45) = .41, *p* = .681 (adolescent/adult *M*=-.23/-.21, *SD* = .26/.21).

### 3.6 ANOVA: blaster preferences

**Fig. 4:**
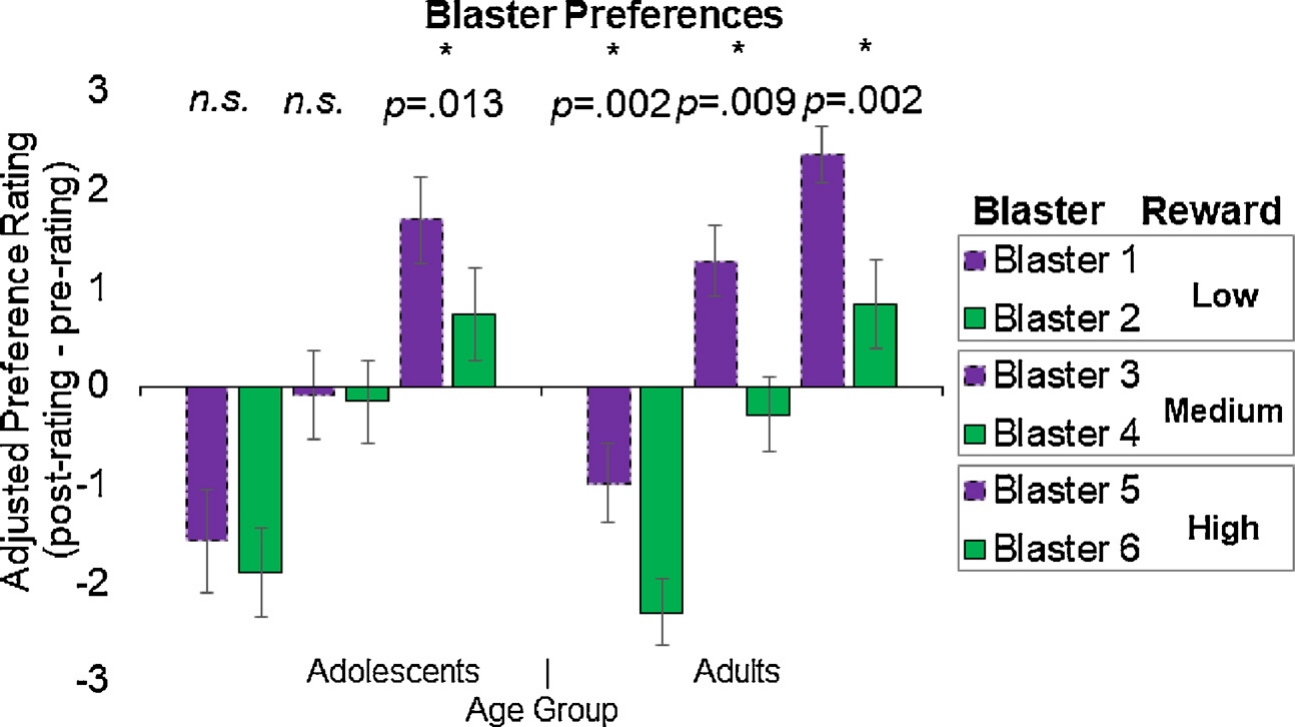
Effect of Effort on Adjusted Preferences. Adjusted preferences represent the degree to which game play increased participants’ preferences for each blaster. Whereas adults consistently preferred the relatively lower-effort options (shown in purple with dashed lines) compared to the relatively higher-effort (shown in green with solid lines), adolescents only differentiated between high and extreme effort levels, which were associated with the highest level of reward. (For interpretation of the references to colour in this figure legend, the reader is referred to the web version of this article).

Adjusted preferences (post preference – pre preference), representing the degree to which game play influenced preferences for each blaster, are displayed in Fig. 4. (For an alternative graphic representation of pre- and post- preference ratings see Supplementary Fig. 1) Positive values represent increases in liking from before to after the experiment, and negative values represent decreases in liking. An ANOVA examining effects of reward, relative effort (whether the blaster was the high or low effort option within each reward level), and age group on adjusted preference revealed significant effects (using Bonferroni-corrected *p* < .025) of reward, *F*(2,45) = 40.21, *p* < .001, ηp2=.641; and relative effort, *F*(1,46) = 15.21, *p* < .001, ηp2=.248; indicating that preferences were informed by both reward and effort in the predicted directions across both age groups. The effect of age group on overall preferences was not significant, *F*(1,46) = 2.29, *p* = .137. While the interaction between relative effort and age group *F*(1,46) = 4.29, *p* = .044, ηp2=.085 was significant at the *p* < .05 threshold and had a medium effect size, it did not reach the Bonferonni correction threshold of *p* < .025.

Whereas adults consistently preferred lower-effort options within each reward level (Blaster 1 > 2, Blaster 3 > 4, Blaster 5 > 6), adolescent preferences only differentiated between the high and extreme effort levels (Blaster 5 > 6; Fig. 4). Furthermore, whereas adults showed positive (above-zero) preference change for the less effortful of the two medium-reward blasters, adolescents remained neutral on the medium-reward, lower-effort blaster, and showed positive preference change only for the two blasters that hit the highest number of aliens. Together, this pattern of results suggests that adolescents exhibited less sensitivity to effort costs than adults, however, as the overall interaction between relative effort and age group did not survive Bonferonni correction, there is a need to interpret these results with some caution.

### 3.7 ANOVA: blaster choices

An ANOVA examining effects of reward, relative effort (high or low effort within each reward level), and age group was also performed on blaster choice. Paralleling the results of the ANOVA on preference ratings, the results for the choice ANOVA revealed significant effects (using Bonferroni-corrected *p* < .025) of reward, *F*(2,45) = 73.35, *p* < .001, ηp2=.765; and relative effort, *F*(1,46) = 14.98, *p* < .001, ηp2=.247. Further paralleling results of the ANOVA on preference ratings, the interaction between relative effort and age group *F*(1,46) = 4.41, *p* = .041, ηp2=.087 was significant at the *p* < .05 threshold and had a medium effect size but did not reach the Bonferonni correction threshold of *p* < .025. As with the preference ratings, relative effort appeared to exert a stronger influence over task choices in adult participants compared to adolescents, however, as this effect did not survive Bonferonni correction, caution is needed when interpreting the effect.

### 3.8 Regression: blaster preferences

Within subjects, multiple regressions were run to quantify the influence of the four levels of effort and three levels of reward on adjusted preferences. For each participant, unstandardized regression coefficients were calculated (from the individual subject-level regressions) and used to compare the two age groups. In the adolescent group, reward exerted a significant (non-zero) effect on adjusted preference controlling for effort, *t*(22) = 3.77, *p* = .001; however, effort controlling for reward did not, *t*(22)=-1.16, *p* = .257. In the adult group, both effort and reward exerted significant effects, with reward exerting a significant positive effect (increasing preferences) *t*(24) = 10.71, *p* < .001, and effort exerting a significant negative effect (diminishing preference), *t*(24)=-4.62, *p* < .001. See also Supplementary Tables 2 and 3 for analogous multilevel model results. T-tests comparing age groups revealed significant differences between adolescents and adults, such that effort exerted a stronger negative effect (diminishing preferences) in the adult age group compared to adolescents, *t*(46) = 2.05, *p* = .046, *d* = .591 (adolescent/adult *M*=-.062/-.203, *SD* = .256/.220), and reward exerted a marginally significant stronger positive effect on increasing preferences in the adult group, *t*(46)=-2.02, *p* = .050, *d*=-.575 (adolescent/adult *M* = 1.91/3.06, *SD* = 2.44/1.43). While between-group effect sizes were medium, the between-group comparisons did not survive the Bonferonni-corrected threshold of *p* < .025, and thus, there is a need to interpret the between-group results with some caution. Multilevel model results (see Supplementary Table 4) mirrored these findings with both effort and reward exerting stronger effects among the adult group. A separate regression revealed that the ratio of reward to effort significantly influenced preferences in both the adolescent and adult groups, *t*(22) = 4.50, *p* < .001; *t*(24) = 10.38, *p* < .001; respectively, and there was no significant effect of reward/effort ratio on blaster preferences between the age groups, *t*(46)=-1.57, *p* = .124, *d*=-.463.

### 3.9 Regression: blaster choices

Paralleling the preference regression, subject-level multiple regressions were run to quantify the influence of the four levels of effort and three levels of reward on choice behavior. The adolescent group exhibited a significant (non-zero) effect of reward on choice behavior controlling for effort, *t*(22) = 5.64, *p* < .001; however, effort controlling for reward did not, *t*(22)=-1.28, *p* = .213. In the adult group, both effort and reward exerted significant effects, with reward exerting a significant positive effect (increasing choice) *t*(24) = 10.60, *p* < .001, and effort exerting a significant negative effect (diminishing number of choices), *t*(24)=-4.17, *p* < .001. See also Supplementary Tables 2 and 3 for analogous multilevel model results. A *t*-test comparing age groups revealed significant differences between adolescents and adults, such that effort exerted a stronger negative effect (diminishing number of choices) in the adult age group compared to adolescents, *t*(46) = 2.07, *p* = .044, *d* = .60 (adolescent/adult *M*=-.06/-.20, *SD* = .23/.24). Again, while the between-group effect size was medium, the effect did not survive the Bonferonni-corrected threshold of *p* < 0.025 and should thus be interpreted with some caution. The effect of reward on increasing choice behavior was stronger in the adult group, but not significantly so, *t*(46)=-1.91, *p* = .062, *d*=-.549 (adolescent/adult *M* = 2.69/3.82, *SD* = 2.29/1.80). Multilevel model results (see Supplementary Table 4) mirrored these findings with both effort and reward exerting stronger effects among the adult group. A separate regression revealed that the ratio of reward to effort significantly influenced choice behavior in both the adolescent and adult groups, *t*(22) = 6.28, *p* < .001; *t*(24) = 11.05, *p* < .001; respectively, and there was no significant age difference in the effect of reward/effort ratio on choices, *t*(46)=-1.41, *p* = .164, *d* = .416.

### 3.10 Correlation: DARS scores with effort and reward coefficients

We performed post-hoc correlations between participants’ overall DARS scores and their effort and reward regression coefficients for two primary reasons: (1) contrary to study hypotheses, we found evidence (marginally significant) that reward exerted a stronger effect on preferences and choices for adults compared to adolescents and correlating a self-report measure of reward functioning with task parameters provides a validity check for task-based measures, and (2) we hypothesized that this unexpected effect of a relatively weak influence of reward among adolescents may be associated with the adolescents’ significantly lower DARS scores, which presumably represent decreased general reward responsivity in the adolescent sample compared to the adult sample. Using unstandardized betas from the preference regression, DARS scores showed a significant negative correlation with effort betas (r(46) = -.31, *p* =  .034) and a non-significant positive correlation with reward betas (r(46) = .197, *p* =  .185). Using unstandardized betas from the choice regression, DARS scores showed a significant negative correlation with effort betas (r(46) = -.349, *p* =  .016) and a significant positive correlation with reward betas (r(46) = .382, *p* =  .008). As two sets of parallel correlation analyses both tested our post-hoc hypothesis, we again used the Bonferonni-corrected threshold of *p* < 0.025; only the correlations with coefficients from the choice regressions survived this correction for multiple comparisons.

## 4 Discussion

The present study introduced a novel, developmentally-appropriate task designed to measure willingness to expend effort in pursuit of a goal and demonstrated that adolescents, compared to adults, consistently exhibited reduced sensitivity to physical effort costs, suggesting that adolescents may be more willing than adults to expend effort to obtain reinforcements of increasing magnitude. Because adolescents and adults were equally accurate in estimating which effort category the blaster options belonged to, it appears that adolescents may be more willing to expend effort, rather than less aware of the exertion. Previous research in adolescent decision making has primarily focused on reward sensitivity, risk assessment, and delay discounting. Thus, the present effort-based decision making study is a novel contribution and offers a promising new direction in adolescent decision research.

Preliminary evidence suggests the alien blasting effort task is a valid tool for measuring effort-based decision making in adolescents and adults. In addition to both age groups correctly estimating the effort categories, rewards used in this task (hitting aliens) appeared to be equally salient to adolescents and adults and no differences were found between the groups in intrinsic motivation for the task. Across multiple analyses, and for both age groups, increasing effort levels had a negative influence, while increasing reward levels had a positive influence on preferences and choice behavior. Further, a self-report measure of reward responsivity (the DARS) positively correlated with reward-related coefficients and negatively correlated with effort-related coefficients, suggesting that real-world reward functioning relates to task-based estimates of the parameters driving effort-based decisions. In line with study hypotheses and across analyses, we found consistent evidence for a stronger negative effect of effort on preferences and choices among adults than among adolescents. However, there was mixed evidence for between-group differences related to the influence of reward. Based on prior work on adolescent decision making (for review see Galvan, 2010), we hypothesized that reward would exert a stronger influence on adolescents. In support of this hypothesis, adolescents only showed positive changes in preference for the two blasters with the highest level of reward, while adult preference changes reflected both effort and reward levels. Further, in both regression analyses, reward, controlling for effort, exerted a positive effect on adolescents, but effort, controlling for reward, did not. In contrast, both factors exerted an effect when controlling for the other in adults. However, contrary to our reward-related hypothesis, when the influence of reward was directly compared between the age groups, reward exerted a stronger influence on adult preferences and choices—although, these differences were only marginally significant (*p*’s in .05--.06 range). It is also important to note here that between-group differences (both for the influence of effort and for reward) did not survive correction for multiple comparisons, suggesting the need to interpret all between-group differences with caution. The results of the multilevel models (see Supplementary Results) supported the primary findings that effort exerted a stronger influence among adults thanamong adolescents.

Another important caveat for interpreting the between-groups effects is the significantly lower reward responsivity (overall DARS scores) reported by the adolescents compared to the adults. Lower self-reported reward responsivity should correspond to reduced influence of reward on decisions. Thus, the adolescent sample’s lower DARS scores may explain the unexpected finding of reward’s stronger influence among adults. On the other hand, lower levels of reward responsivity should align with effort exerting a stronger negative effect (Geaney et al., 2015; Treadway, et al., 2009). This lends strength to the finding that effort indeed had a weaker effect on adolescents, as their lower DARS scores would, if anything, be associated with an increase in effort’s influence. For a more definitive test of effort and reward’s influence across different developmental cohorts, future work should utilize higher powered samples and ensure groups are better matched in terms of self-reported reward responsivity.

The alien blasting effort task offers researchers in developmental neuroscience a novel tool for exploring changes in the neural circuitry underlying effort-based decisions across development. Cost-benefit decisions, like decisions to exert effort for a desired outcome, entail communication between “reward processing” in the mesolimbic system and “cost evaluation” instantiated in higher order cortical structures (Arulpragasam et al., 2018; Bailey et al., 2016; Floresco et al., 2008; Shenhav et al., 2017). Dopamine signals projecting along the mesolimbic pathway to the striatum are known to represent reward value, which guides effort-based decisions. As such, striatal dopamine plays a major role in motivating effortful behavior, facilitating the exertion of greater effort to pursue higher-value outcomes (Assadi et al., 2009; Floresco et al., 2008; Hosking et al., 2015; Salamone et al., 2016b). Rodent studies have demonstrated that surgical and pharmacological interference with dopamine transmission greatly impairs effort-based decision making by reducing effort exertion, while leaving reward enjoyment and motor abilities intact (Floresco et al., 2008; Salamone et al., 2016a), and human research utilizing PET imaging has revealed that willingness to exert effort correlates positively with dopamine functioning in the adult striatum (Treadway et al., 2012). While neural structures within the reward system such as the ventral striatum and ventromedial prefrontal cortex represent the net value of exerting effort to achieve reward, cortical regions such as the dorsal anterior cingulate cortex and anterior insula are involved in higher-level integrative processes that incorporate effort cost-related signals into net value representations (Arulpragasam et al., 2018; Botvinick et al., 2009; Chong et al., 2017; Croxson et al., 2009; Shenhav et al., 2017).

To date, relatively little is known about effort-based decision making in adolescents compared to risk-based decision making in this age group. However, there are likely important parallels between these two domains. Both types of decision making involve tradeoffs between rewards and costs (potential risk, effort). As such, adolescent risk-based decision making may provide a corollary for effort-based decision making in adolescents. The dual systems account of adolescent risk-taking asserts that adolescents are strongly biased by reward in the context of risky decision making because reward circuitry develops along an inverted U-shaped trajectory that peaks in adolescence while higher-order cognitive control circuitry, which supports the calculation of risk, develops linearly with age (Casey et al., 2008; Cohen, 2005; Steinberg, 2008; van Leijenhorst et al., 2010a). Thus, adolescent reward circuitry is hyper-responsive (Galvan, 2010) causing adolescents to weight decisions in favor of potential rewards over potential risks. As effort costs are also evaluated within adolescent’s relatively immature cortical regions, effort costs, like risk costs, may be weighted less heavily among adolescent decision makers.

Supporting this correspondence across different domains of adolescent decision making, research has demonstrated that development within the corticostriatal reward system has been linked to adolescent changes in, not only risk-based decisions (Casey, 2015; Galvan et al., 2007), but also delay-based decisions (Christakou et al., 2011; van den Bos et al., 2015). Across risk- and delay-based decision making domains, heightened activity/ connectivity of reward centers is associated with decision biases towards reward, while maturation-associated increased connectivity between reward centers and cortical regions predicts greater tolerance for delay costs. It has been suggested that the adolescent bias for reward (and associated tolerance for risk/uncertainty/ impatience for delay) might be advantageous inasmuch as these developmental patterns contribute to new experiences that can help to scaffold adolescents’ burgeoning independence (Hartley and Somerville, 2015). While developmental changes in neurobiology have not yet been linked to adolescent decision making in an effort context, it stands to reason that greater willingness to expend effort might be similarly adaptive during adolescence, allowing young people to gain new experiences and insights. Because the adolescent reward system is primed to respond strongly to rewarding outcomes, we predicted that adolescents might show a corresponding increase in willingness to exert effort in service of their goals. While evidence for increased responsivity to reward among adolescents was mixed in the current study, we found consistent evidence for increased tolerance for effort costs among adolescents compared to adults.

### 4.1 Limitations

The present study was designed to examine whether adolescents may exhibit increased willingness to exert effort in pursuit of a goal relative to adults. Because the pilot sample is relatively small, findings should be considered tentative. We have found preliminary evidence for age differences in the effects of effort on both explicit preferences and choices made during the game, however, these effects, while consistent across multiple analyses, did not survive correction for multiple comparisons and should therefore be interpreted with caution and replication in a larger sample is needed. A second limitation is related to preferences for effort expenditure versus perceiving/learning about differences in effort levels. Although we did ask participants to categorize the blasters’ effort levels and found no differences among the age groups, this is a relatively blunt measure of participants’ perceptions of effort levels. Thus, it is impossible to fully distinguish whether adolescents are truly less sensitive to effort costs or are simply less adept at perceiving or learning about effort costs. Future work could resolve this issue by better measuring the accuracy of effort perception/learning by asking participants to estimate the average number of presses associated with each effort stimulus. A third limitation relates to the correlation between reward magnitude and reward rate (reward/effort ratio). Reward magnitude and the magnitude of cost (effort cost in the current study) are generally correlated across different types of cost-benefit decision making tasks (e.g., Treadway et al., 2009; McClure et al., 2004) for the sake of external task validity. However, reward magnitude can be uncorrelated with reward rate (reward/effort ratio) as a way to control for potential differences in the optimality of an effort-avoiding versus reward-seeking task strategy. To uncorrelate reward magnitude and reward rate, stimuli must include effort/reward pairings at more extreme ends of optimality—that is, the task would have to offer both very sub-optimal options (many presses for reward) and extremely optimal options (very few presses for reward). The current study focused on less extreme effort/reward stimuli pairings under the assumption that options of similar optimality would elicit maximum individual differences (because there would be little variation in individual’s choices for very sub-optimal and extremely optimal options). However, in the future, researchers may want to better control for potential differences in task outcomes based on whether participants adopt an effort-avoiding versus reward-seeking task strategy. We have therefore included in the Supplemental Methods a hypothetical distribution of effort/reward pairings for stimuli that could be used with this task in order to uncorrelate reward magnitude and reward rate (see Supp Table 1). Finally, an additional limitation concerns the age of the adult sample, which included participants ages 18-23. Although this age range is commonly characterized as adult in non-developmental research, the field of adolescent development has increasingly moved toward a definition of adolescence that extends into the early twenties (Sawyer et al., 2018). Therefore, we believe it will be of great importance to administer this task to a wider age range to ascertain whether the pattern of greater sensitivity to effort costs observed in the adult sample would replicate in an older group of adults.

### 4.2 Future directions

In addition to replicating the current results in a larger sample with developmental groups better matched in self-reported reward responsivity and extending the current work to include additional epochs of development, future work could also use the alien blasting task to explore other research questions related to the effects of reward and effort, particularly as this task lends itself well to flexible manipulations of both reward and effort. For example, this task could be made more difficult (by decreasing the press rate and latency multipliers from the calibration task) and task-related rewards (aliens hit) could be traded in for age-appropriate prizes to enhance the salience of reward (Hawes et al., 2017). This task could also be used to explore effort/ reward tradeoffs for different classes of rewards. For example, on different rounds of the task, participants could be instructed to compete with their own previous score or to compete with a peer’s score, as a way to compare intrinsic versus social reward (Dimenichi and Tricomi, 2015). Additionally, one interesting question, alluded to above, is whether potential age-related differences in ability to explicitly perceive or learn about levels of effort might affect effort-based preferences and choices.

The present study validated a novel task that shows great promise for examining effort-based decision making during adolescence and beyond. Effort-based decision making is an understudied but exciting avenue for developmental research. Learning new skills and acquiring new knowledge bases are crucial steps on the path to independence, which could be greatly facilitated by the willingness to engage in effortful pursuit of new experiences and goals. Adolescence is already viewed as a time of increased risk taking and reduced tolerance for delayed gratification; these findings suggest that it may also be a time of increased willingness to expend effort during goal pursuit. Exploiting this increased willingness to expend effort may be an untapped means to achieve greater engagement in teens.

## Funding

This project was supported by funding from the National Science Foundation [Postdoctoral Fellowship 1606979 to S.D.] from the University of California Consortium on the Developmental Science of Adolescence [seed grant to S.D.] and the Jeffrey/Wenzel Term Chair to AG.
