# Supplementary material for: Worth working for: The influence of effort costs on teens’ choices during a novel decision making game
Source: Dev Cogn Neurosci. 2019 Apr 30;37:100652. doi: 10.1016/j.dcn.2019.100652 (PMC6969283; doi:10.1016/j.dcn.2019.100652)
Supplement: Supplementary file 1 [file mmc1.docx]

**Supplementary Material**

**Supplementary Methods**

**Uncoupling Reward Magnitude and Reward Rate in Task Stimuli**

Table S1: Actual and Hypothetical Distributions for Reward and Effort Pairings
within Stimuli

The distribution of reward and effort pairings used across stimuli in the current study is presented in chart A. The stimuli used in the current study correlated reward magnitude with both effort level and reward/effort ratio (reward rate). Chart B provides a hypothetical distribution for stimuli reward/effort pairings in which reward magnitude and reward rate are uncorrelated (although reward magnitude and effort level remain correlated). While reward magnitude and costs (effort level in the current study) are generally correlated in cost/benefit decision making tasks (e.g., Treadway, et al., 2009; McClure, et al., 2004), future users of this task may want to uncouple reward magnitude and *reward/effort ratio* (reward rate), as a way to control for potential differences in the optimality of effort-avoiding versus reward-seeking task strategies. In order to reduce the total number of choice trials required, a smaller subset of the stimuli in chart B could be used by excluding all of the lowest or all of the highest ratios within each reward magnitude.

**Multilevel Models of the Influence of Reward, Effort, and Age Group**

Multilevel models were conducted using Linear Mixed-Effects (LME) models (lme4 in R: Bates et al., 2015) in order to verify main results. The first set of models were run separately for adolescents (Supplementary Table 2) and adults (Supplementary Table 3) and examined the effects of Reward (3 levels) and Effort (4 levels) on two dependent variables: (A) Adjusted Preferences and (B) Number of Times Chosen. This set of models was also run collapsing across the age groups (Supplementary Table 4). Age group effects were added in the second set of models, with input variables of Age Group (adult as reference group) and two interaction terms, Reward X Age Group and Effort X Age Group (Supplementary Table 4).

**Supplementary Results**

**Blaster Preferences**

Figure S1: Pre to Post Blaster Preferences for Adolescents and Adults

**Multilevel Modeling Results**

Table S2: Multilevel Model within Adolescent Group

Table S3: Multilevel Model within Adult Group

Table S4: Multilevel Model across Age Groups

**Supplementary References**

Bates, D., Maechler, M., Bolker, B., Walker, S. (2015). Fitting Linear Mixed-Effects Models Using lme4. Journal of Statistical Software, 67(1), 1-48.<[doi:10.18637/jss.v067.i01](https://doi.org/10.18637/jss.v067.i01)>.
